# Electronic Health Program to Empower Patients in Returning to Normal Activities After General Surgical and Gynecological Procedures: Intervention Mapping as a Useful Method for Further Development

**DOI:** 10.2196/jmir.9938

**Published:** 2019-02-06

**Authors:** Chantal M den Bakker, Frederieke G Schaafsma, Eva van der Meij, Wilhelmus JHJ Meijerink, Baukje van den Heuvel, Astrid H Baan, Paul HP Davids, Petrus C Scholten, Suzan van der Meij, W Marchien van Baal, Annette D van Dalsen, Daniel J Lips, Jan Willem van der Steeg, Wouter KG Leclercq, Peggy MAJ Geomini, Esther CJ Consten, Steven E Schraffordt Koops, Steve MM de Castro, Paul JM van Kesteren, Huib A Cense, Hein BAC Stockmann, A Dorien ten Cate, Hendrik J Bonjer, Judith AF Huirne, Johannes R Anema

**Affiliations:** 1 Amsterdam Public Health Research Institute Department of Occupational and Public Health VU University Medical Center Amsterdam Netherlands; 2 Department of Surgery VU University Medical Center Amsterdam Netherlands; 3 Department of Obstetrics and Gynaecology VU University Medical Center Amsterdam Netherlands; 4 Department of Operation Rooms Radboud University Medical Center Nijmegen Netherlands; 5 Department of Surgery Amstelland Ziekenhuis Amstelveen Netherlands; 6 Department of Surgery Diakonessenhuis Utrecht Netherlands; 7 Department of Obstetrics and Gynaecology Diakonessenhuis Utrecht Netherlands; 8 Department of Surgery Flevoziekenhuis Almere Netherlands; 9 Department of Obstetrics and Gynaecology Flevoziekenhuis Almere Netherlands; 10 Department of Surgery Isala Zwolle Netherlands; 11 Department of Surgery Jeroen Bosch Ziekenhuis 's-Hertogenbosch Netherlands; 12 Department of Obstetrics and Gynaecology Jeroen Bosch Ziekenhuis 's-Hertogenbosch Netherlands; 13 Department of Surgery Máxima Medisch Centrum Veldhoven Netherlands; 14 Department of Obstetrics and Gynaecology Máxima Medisch Centrum Veldhoven Netherlands; 15 Department of Surgery Meander Medisch Centrum Amersfoort Netherlands; 16 Department of Obstetrics and Gynaecology Meander Medisch Centrum Amersfoort Netherlands; 17 Department of Surgery Onze Lieve Vrouwe Gasthuis Amsterdam Netherlands; 18 Department of Obstetrics and Gynaecology Onze Lieve Vrouwe Gasthuis Amsterdam Netherlands; 19 Department of Surgery Rode Kruis Ziekenhuis Beverwijk Netherlands; 20 Department of Surgery Spaarne Gasthuis Hoofddorp Netherlands; 21 Department of Obstetrics and Gynaecology Spaarne Gasthuis Haarlem Netherlands

**Keywords:** intervention mapping, eHealth, return to normal activities, return to work, patient reported outcome measures, colectomy, hysterectomy

## Abstract

**Background:**

Support for guiding and monitoring postoperative recovery and resumption of activities is usually not provided to patients after discharge from the hospital. Therefore, a perioperative electronic health (eHealth) intervention (“ikherstel” intervention or “I recover” intervention) was developed to empower gynecological patients during the perioperative period. This eHealth intervention requires a need for further development for patients who will undergo various types of general surgical and gynecological procedures.

**Objective:**

This study aimed to further develop the “ikherstel” eHealth intervention using Intervention Mapping (IM) to fit a broader patient population.

**Methods:**

The IM protocol was used to guide further development of the “ikherstel” intervention. First, patients’ needs were identified using (1) the information of a process evaluation of the earlier performed “ikherstel” study, (2) a review of the literature, (3) a survey study, and (4) focus group discussions (FGDs) among stakeholders. Next, program outcomes and change objectives were defined. Third, behavior change theories and practical tools were selected for the intervention program. Finally, an implementation and evaluation plan was developed.

**Results:**

The outcome for an eHealth intervention tool for patients recovering from abdominal general surgical and gynecological procedures was redefined as “achieving earlier recovery including return to normal activities and work.” The Attitude-Social Influence-Self-Efficacy model was used as a theoretical framework to transform personal and external determinants into change objectives of personal behavior. The knowledge gathered by needs assessment and using the theoretical framework in the preparatory steps of the IM protocol resulted in additional tools. A mobile app, an activity tracker, and an electronic consultation (eConsult) will be incorporated in the further developed eHealth intervention. This intervention will be evaluated in a multicenter, single-blinded randomized controlled trial with 18 departments in 11 participating hospitals in the Netherlands.

**Conclusions:**

The intervention is extended to patients undergoing general surgical procedures and for malignant indications. New intervention tools such as a mobile app, an activity tracker, and an eConsult were developed.

**Trial Registration:**

Netherlands Trial Registry NTR5686; http://www.trialregister.nl/trialreg/admin/rctview.asp?TC=5686

## Introduction

### Background

The length of in-hospital stay after general surgical and gynecological procedures has decreased significantly due to a growing trend in day-care surgery, introduction of minimal invasive techniques, and enhanced recovery after surgery programs (ERAS) [1-3]. Due to this shortening of in-hospital stay, perioperative in-hospital care has been reduced and the greater part of the recovery period takes place at home [4,5]. As a result, guiding and monitoring of resumption of normal activities (RNA) including return to work (RTW) and long-term recovery are now transferred to primary care [6]. However, frequently no or conflicting advice is given, resulting in patients being unsure whom to contact for support in case of complaints. Patients often lack the knowledge themselves to determine how and when to resume activities [7-10]. As a consequence, full recovery after surgery takes much longer than expected despite improved surgical treatment. A longer recovery at home could result in diminished general and mental health, higher medical consumption, lower quality of life, and longer sick leave period [10-13].

Electronic health (eHealth) can be a suitable tool to optimize the quality of perioperative care of patients who will undergo general surgical and gynecological procedures. eHealth can provide tailored information, increase patients’ self-management, and has interactive communication features [14]. Furthermore, it has the potential to empower patients, to motivate patients, and turn them into more active and effective managers of their own health [15-17]. A recovery-oriented eHealth intervention (“ikherstel” intervention or “I recover” intervention) has already proven to be effective with a significant faster RTW after benign gynecological surgical procedures [18]. This care program requires a need for further development to fit a broader population of patients who will undergo various types of general surgical and gynecological procedures.

### Objectives

In this paper, the further development with corresponding process of this “ikherstel” intervention is described [19]. The objective of the eHealth intervention development includes to further optimize (1) empowerment of general surgical and gynecological patients during the perioperative period to RNA and RTW and (2) partial substitution of perioperative care with eHealth. For this, the Intervention Mapping (IM) protocol is used, which is a suitable systematic and scientifically accepted method for the (further) development and implementation of a wide range of eHealth and RTW interventions. This method is based on theory and stakeholders’ (including patients’) involvement [20,21].

## Methods

### Overview

**Figure 1:**
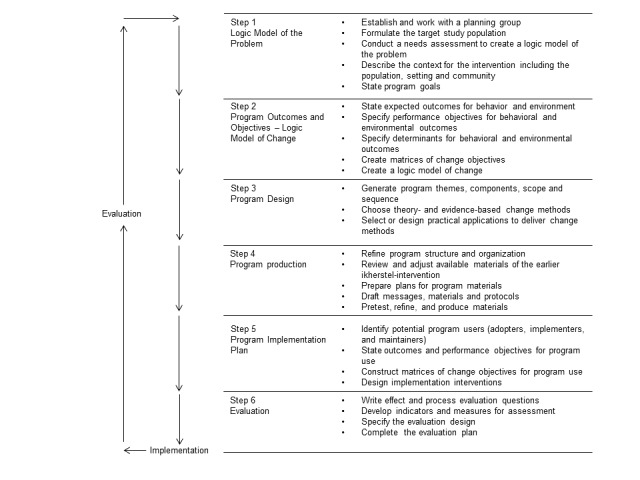
Intervention mapping steps.

The IM protocol for the further development of the “ikherstel” intervention consists of 6 steps (see Figure 1): (1) forming a logic model of the problem; (2) defining program outcomes and objectives; (3) designing the eHealth program; (4) producing the eHealth program; (5) developing a program implementation plan; and (6) making an evaluation plan [20,21].

In the previous “ikherstel” study, only women were included as the intervention was only available for patients undergoing gynecological abdominal procedures. In this study, men will also be included because the target population includes patients undergoing general surgical procedures. Currently, the most performed general surgical abdominal procedures in elective setting in the Netherlands are hernia inguinal repair (with 28,232 procedures per year), cholecystectomy (25,203 procedures), and colectomy (14,012 procedures) [22]. Patients undergoing these surgical procedures are chosen as part of the target population, next to those patients undergoing gynecological procedures including hysterectomy and adnexal surgery as the other part of the target population. This way, a broad patient population of general surgical and gynecological patients can be achieved [18,19,23,24]. The study population is divided in (1) the minor abdominal general surgical and gynecological procedures group, which consists of laparoscopic cholecystectomy and adnexal surgery and laparoscopic or open hernia inguinal repair and (2) the major abdominal general surgical and gynecological procedures group, which consists of laparoscopic or open colectomy and hysterectomy.

In the first 3 preparatory steps of further development, the process is described, starting from, and based on, the original developed “ikherstel” intervention [19]. This original intervention is extended, optimized, and made applicable to the broader target population. In the last 3 steps of the intervention development, the implementation and evaluation plan is described for only the major abdominal general surgical and gynecological procedures group, because the protocol of the randomized controlled trial (RCT) of the minor abdominal procedures is already published elsewhere [25].

### Step 1: Logic Model of the Problem

First, a planning group was composed to work with during the whole IM process. Next, a multifactorial patients’ needs assessment was conducted which included (1) a process evaluation of the earlier performed “ikherstel” study, (2) a review of the literature, (3) a survey study, and (4) focus group discussion (FGDs). Findings from the needs assessment were then compiled into a PRECEDE-PROCEED model (predisposing, reinforcing, and enabling constructs in educational diagnosis and evaluation-policy, regulatory, and organizational constructs in educational and environmental development) to identify the factors the program should address to help improve full recovery including RNA and RTW after surgery at home for abdominal general surgical and gynecological patients in the Netherlands.

#### Process Evaluation

A process evaluation was performed on the previous “ikherstel” intervention to gain more insights into the facilitators and barriers for the acceptance and implementation of this intervention [26]. These results were used for the further development of the new “ikherstel” intervention. We evaluated how the eHealth intervention was delivered to and received by participants and how participants and health care professionals had appreciated the intervention.

#### Review of the Literature

A systematic review was performed to evaluate the effect of perioperative eHealth interventions on the postoperative course of any surgical procedure [27]. This review provides us important and general information about effective aspects of eHealth interventions for the further development of our eHealth intervention.

Furthermore, a literature search was performed to further investigate and gather more information about the average duration of full recovery including RNA and RTW after abdominal surgical procedures including factors that affect this duration. The literature search of the IM procedure of the previous “ikherstel” intervention was used and broadened to identify behavioral and environmental conditions of prolonged sick leave and delayed RTW among gynecological patients [19]. The search was executed in PubMed with the key words “abdominal surger*” and “recover*” or “rehabilitation” or “return to normal activit*” to select articles describing additional factors for delayed RNA in general surgical patients. This also included a search for behavioral and environmental explanations for delayed full recovery.

#### Survey Study

A survey study was performed to explore specific needs in the target population regarding the “ikherstel” intervention. Patients who underwent surgery (minor and major abdominal general surgical or gynecological procedures) between August 2013 and August 2014 in the VU University medical center (located in Amsterdam) received a questionnaire. This survey study was performed to (1) evaluate shortcomings in information and guidance supplied to patients in current perioperative care for patients undergoing general surgical and gynecological procedures, (2) investigate whether eHealth may be of assistance in this, and (3) to identify gender-specific needs [28]. On the basis of these results, the previous “ikherstel” intervention could be further adapted to this population.

#### Focus Group Discussions

In total, 4 FGDs were performed to gather patients’ experiences with recovery after (multimodal) cancer treatment and to identify particular needs during the various phases of cancer treatment. The second objective was to evaluate possible solutions for unmet needs by the introduction of eHealth. By gathering this information, the intervention can be adjusted in such a way that it is also applicable to patients undergoing surgery due to a malignant indication. Colon cancer patients were recruited from patient files of 2 Dutch teaching hospitals: Meander Medical Center (located in Amersfoort) and Spaarne Gasthuis hospital (located in Hoofddorp and in Haarlem). Endometrial cancer patients were recruited from the patient files of 3 teaching hospitals: VU University medical center (located in Amsterdam), Antoni van Leeuwenhoek hospital (located in Amsterdam), and the Flevo Hospital (located in Almere).

### Step 2: Program Outcomes and Objectives-Logic Model of Change

In the second step, the program outcomes and objectives were developed. Performance objectives were specified to describe in detail the patients’ behavioral and environmental outcomes that were considered necessary to reach full recovery after surgery. Determinants for these behavioral and environmental outcomes were selected, and by crossing the performance objectives with these determinants (constructing matrices), the change objectives were identified. These matrices were used to identify behaviors and conditions that result in a sustainable full recovery. This step aimed to determine whether performance objectives, behavioral determinants, and change objectives of the earlier “ikherstel” intervention needed to be modified to better fit the broader target population. A selection of the performance objectives of the IM article of Vonk Noordegraaf et al was further supplemented with new performance objectives based on the findings of the needs assessment and the literature on behavior change [19].

### Step 3: Program Design

In the third step, program themes, components, scope, and sequence were specified and theory- and evidence-based change methods were searched in PubMed and applied based on the findings in the first 2 steps. Theory-based methods for change are general techniques or processes that have been shown to enable change in one or more determinants of behavior and have their origins in behavioral and social science theories [20,29]. Choosing the theory- and evidence-based change methods and selecting or designing practical applications to deliver change methods was done by the planning group and guided by the formulated performance and change objectives in the second step of the IM protocol and were also based on the performance and change objectives as described in the earlier performed “ikherstel” study [19].

### Step 4: Program Production

In the fourth step, the program structure was refined and organization was planned. All gathered information from the previous steps was synthesized and translated into plans for drafting program materials, that is, tailored tools and information to empower major abdominal general surgical and gynecological patients by an innovative eHealth care program. Participants of the FGDs in step 1 and health care professionals (surgeons, gynecologists, and residents in training) outside the project group pretested the different aspects of our eHealth intervention, whereby possible bugs and shortcomings were identified, refined, and adjusted.

### Step 5: Program Implementation Plan

In the fifth step, potential program users were reidentified in line with the broadening of the patient population and the consequences for the implementation and acceptance of the eHealth intervention. With this information, an implementation plan to enable an extensive evaluation of the intervention was developed including a plan to identify and inform the health care professionals and researchers in the participating hospitals about the different core components of the intervention. Furthermore, details about optimal delivery of the intervention were incorporated herein. Hospitals that participated in previous “ikherstel” studies gave their approval to participate again. Other hospitals were recruited by the PhD candidates. The planning of the adoption and implementation of the intervention was established.

### Step 6: Evaluation

In the sixth step, an evaluation plan of the intervention was developed, which involves determining whether behavior outcomes change as a result of the adjusted eHealth intervention. For this evaluation, an RCT was designed to measure the effects of the eHealth intervention on RNA and RTW for patients undergoing major abdominal general surgical and gynecological procedures. This study was approved by the Medical Ethics Committee of the VU University medical center under registration number 2014.301. This study was also registered at the Netherlands Trial Registry under registration number NTR5686.

## Results

### Step 1: Logic Model of the Problem

The planning group (development team) consisted of 2 PhD candidates, 2 occupational health physicians, 3 surgeons, and 1 gynecologist. This multidisciplinary planning group was established to further develop the intervention by applying the various steps of the IM process. The members were selected to represent various fields of expertise required for the design of the intervention.

#### Process Evaluation

In the earlier performed “ikherstel” study, 210 patients were included, of which 110 patients were allocated to the intervention group. The implementation score of the intervention was 80%. All patients were given access to the “ikherstel” intervention and 86.4% made a convalescence plan. Reasons for not making or not adhering to the convalescence plan include patients (1) preferred to resume activities when they felt ready for it, (2) found the convalescence plan too optimistic or too conservative, (3) felt pushed by the convalescence plan, and (4) felt that the plan did not apply to their personal situation.

The eHealth intervention was perceived effective by 74% of patients, and 76% of involved gynecologists were satisfied with the Web portal. In total, 95% of the health care professionals would offer the intervention to their patients in the future, and 85.3% of patients would recommend the “ikherstel” eHealth program to a friend.

Required access to internet, the inflexibility of the eHealth intervention in case of complications for patients, and an increased time investment for gynecologists were rated as possible future usage barriers. Suggestions for improvement included an extra section with experiences of other women. All results of the process evaluation are published in detail elsewhere [26].

#### Review of the Literature

The systematic review included 27 studies that focused on replacing or complementing perioperative usual care with some form of care via information and communication technology (ICT) such as telemonitoring, telerehabilitation, teleconsultation, or an educational or supportive website in various types of surgery. Of these studies, 92% reported at least an equal or positive effect of the eHealth intervention compared with usual care on patient-related outcomes. The results show that eHealth or other forms of ICT and telehealth improve clinical outcomes, knowledge, and satisfaction of patients undergoing various forms of surgery. Furthermore, eHealth improves RTW and daily functioning after surgery. Positive influencing factors on these patient-related outcomes were (1) easy access to the intervention, (2) expectation management, and (3) a combined symptom monitoring by blended care [27].

The literature search in the IM protocol article of Vonk Noordegraaf et al identified that pain and discomfort, feelings of fear, and infections were delaying factors for RTW [19]. In addition, literature showed that the substantial variation in convalescence recommendations given by health care professionals were also of influence on the total duration of sickness absence [9,30]. In the broadened search, a wide variety in convalescence duration after minor general surgical procedures (eg, cholecystectomy and hernia inguinal repairs) was observed [31,32]. For major abdominal procedures, limited research was available to analyze the time of full recovery including RNA (and RTW) [33-36]. However, literature does show that the total duration of convalescence was longer than expected despite the implementation of minimal invasive techniques and ERAS programs applied by major abdominal procedures [1,37,38]. Duration of convalescence depends on pain, complications, or fear of recurrence (in case of cancer of hernia inguinal repairs) [39-41]. Preoperative expectations of convalescence, size of the incision, and fatigue were important contributory factors to explain actual convalescence [39-42]. Furthermore, patients who were physically active after colorectal surgery were more likely to recover faster [43].

#### Survey Study

In total, 57.2% (207/362) potential participants completed the survey. Mean age of participants was 46.6 years and almost 30% were male. For 87.4% participants, the indication for surgery was benign with an equal distribution between general surgical and gynecological procedures.

A reported shortcoming related to information and guidance provision was the lack of detailed advice about the resumption of activities following surgery. Many participants reported receiving inconsistent recommendations from medical specialists, general practitioners, and occupational physicians. Limited guidance from professionals during the recovery process was also mentioned as a shortcoming by 40% of the participants. Some participants preferred to receive more information or more emotional and mental support after their surgical procedure. A perceived lack of information or support resulted in more nervousness before surgery or insecurity after surgery.

eHealth was expected to be a very useful tool to overcome these above-mentioned shortcomings. If an eHealth intervention had been available before or after their surgical procedure, 71% of the participants reported they would have used it. Most popular rated items of a future eHealth care program were a page containing an overview of important telephone numbers, a list with frequently asked questions, and the possibility to self-evaluate symptoms after surgery. Furthermore, the option of an electronic consultation (eConsult) was rated useful by 57.6%, and almost half of the participants preferred to use the “ikherstel” intervention also via a mobile phone app. The option to give an employer or an occupational physician access to parts of the website and the option of a patient forum were not rated useful. Limited gender differences in preferences were identified in this survey. Women showed a slightly higher need for information and preferred some extra eHealth support. The total results of the survey study are published elsewhere [28].

#### Focus Group Discussions

For this study, 40% (30/75) potential participants were willing and available to participate in the colon cancer FGDs. For the endometrial cancer FGDs, 35% (17/48) potential participants were willing to participate. A total of 22 patients actually participated in the colon cancer FGDs and 12 in the endometrial cancer FGDs. Most frequently reported unmet needs in the perioperative phase of colon cancer patients were an absence of tailored, dosed, and understandable information and advice regarding RNA. Colon cancer patients who had finished the adjuvant chemotherapy phase would have liked to receive more information about side effects, more mental support, and a longer aftercare period. Endometrial cancer patients evaluated the received information and guidance as very well. This was mainly due to very good guidance from the gynecologist and physician assistant. The participants without adjuvant treatment reported that they would like to have more recommendations regarding resuming normal activities, and they saw a role for eHealth to support this. However, patients who had adjuvant treatment rated the nurses’ guidance as sufficient and needed no additional support.

Colon cancer patients treated with multimodal treatment and endometrial cancer patients who did not receive any adjuvant treatment reported that eHealth services could be supportive but not a substitute of personal interaction with health care professionals. There was a preference for a Web-based health care system that is readily available 24/7 in the form of blended care. As there is already a lot of information on the internet about cancer diagnoses and treatment, it was sometimes hard for participants to differentiate which information is correct. Recommendation of the tool by their own health care professional would enhance perception of safety and, therefore, increase usage. In contrast, endometrial cancer patients who received adjuvant treatment did not see an added value for eHealth. This difference might be explained by the age difference in these patient groups. Results of the FGDs will be reported in more detail as separate papers.

On the basis of the results of this needs assessment, the overall desired outcome for further development of the “ikherstel” intervention tool was defined as “achieving earlier recovery including RNA and RTW.” Furthermore, the findings from the needs assessment were compiled into a PRECEDE-PROCEED model to identify patients’ problems and needs in perioperative surgical care in which eHealth and mobile health (mHealth) can have a (complementary) role and are defined as:

- A lack of clear and simple instructions for the RNA-including work;
- Inconsistent recommendations from different health care providers;
- A lack of information about surgical procedures and the perioperative course, symptoms, and complications;
- Limited mental support in case of patients with a malignant indication;
- Delayed and limited mobilization interventions to realize earlier RNA and RTW; and
- No or limited interaction with their health care professional during postoperative course.

### Step 2: Program Outcomes and Objectives-Logic Model of Change

In the previous “ikherstel” intervention, the following overall objectives were formulated. After the needs assessment, these objectives were adjusted to align to patients’ problems and needs as identified under step 1:

- To enhance recovery by giving clear and simple instructions for the RNA;
- To stop inconsistent recommendations from different health care providers;
- To take away the insecurity with respect to postoperative course, symptoms, and complications.

The following overall objectives were added to the existing objectives after the needs assessment in step 1:

- To provide extra attention and mental support in case of patients with a malignant indication,
- To encourage patients to a quicker and more intense mobilization and earlier RNA, and
- To let patients have more interaction with their health care professional via a web portal.

Specified performance objectives for the further development of the “ikherstel” intervention are presented in Multimedia Appendix 1. To create a matrix of *performance objectives*, the main personal and external determinants of behavior change for each performance objective were operationalized. The Attitude-Social Influence-Self-Efficacy model was used in the IM procedure of the earlier “ikherstel” intervention and was used for the further development of this intervention as well [19]. This model was still considered by the planning group as the most suitable model for recovery and behavior change and, thereby, used to form change objectives [44-49]. Skills, barriers, and facilitators were considered relevant factors for RNA [50,51]. In Multimedia Appendix 2, an example of the performance objective “mobilize quickly and more intense after operation” is presented.

### Step 3: Program Design

The same practical methods and suitable strategies as in the IM article of Vonk Noordegraaf et al were used for the further development of tools and materials of the “ikherstel” intervention as the methods and strategies used in this intervention already had been proven effective [19,52]. For example, self-monitoring of behavior (awareness) and getting direct feedback will be used to stimulate patients to mobilize quickly and more intensively. An activity tracker will be used in the “ikherstel” intervention to encourage this. Multimedia Appendix 3 presents more examples of these methods with preconditions and final tool/materials of the eHealth intervention.

### Step 4: Program Production

The knowledge gathered in the first 3 steps was discussed by the planning group at several meetings to add various appropriate tools to the existing “ikherstel” intervention. A designer/developer specialized in eHealth and mHealth interventions was consulted during some of the meetings. In addition, experienced surgeons, gynecologists, and residents in training outside the planning group were consulted to judge the medical content of the tools. The words “ikherstel.nl” in the internet address of the eHealth intervention means “I am recovering.” The mobile app was made available for iOS and Android mobile phones and tablets. The design of the further developed “ikherstel” intervention for patients undergoing major abdominal surgical and gynecological procedures (this includes open or laparoscopic colectomy and open or laparoscopic hysterectomy) is described below. In Table 1, an overview of all tools of the developed eHealth intervention is presented.

#### Adaptation of Existing “ikherstel” Intervention Tools

##### Website

The website aims to prepare patients in the best possible manner for their surgery and to offer guidance during their recovery process until full recovery and resumption of all activities are achieved. The following tools on the website support this.

**Table 1:** Component of the “ikherstel” intervention regarding each target population.

Tool		Content	Target population
**Website**			
	Information by text and animations about the surgical procedure	Enhancing patient preparation including creating expectations	All participants
	Personalized convalescence plan	Enhancing patient preparation including creating expectations	All participants
	Recovery monitor and recovery report	Monitoring recovery and offering assistance when relevant	All participants
	Video	Increasing the information provision by using several ways to provide this	All participants
	Glossary	Increasing the information provision by using several ways to provide this	All participants
	Frequently asked questions	Increasing the information provision by using several ways to provide this	All participants
	Electronic consultation	Increasing access to care and reducing patient uncertainties and fear related to the recovery process and workload	All participants
	Information about malignancies	Reducing anxiety and uncertainty and increasing the amount of information provision	All participants with malignant disease
	Information about chemotherapy and side effects	Reducing anxiety and uncertainty and increasing the amount of information provision	All participants with malignant disease
	Links for supportive care needs	Creating long-term support to reduce anxiety and uncertainty	All participants with malignant disease
**Mobile app**			
	Information by text	Enhancing patient involvement and recovery expectations and reducing anxiety	All participants with a smartphone
	Insight into the convalescence plan	Creating recovery expectations and improving recovery	All participants with a smartphone
	Recovery monitor and recovery report	Reducing uncertainties and fear related to the recovery process and improving monitoring and transition of postoperative care	All participants with a smartphone
	Creating a packing list	Increasing better patient preparation before admission	All participants with a smartphone
	Section to make notes	Increasing better patient preparation before admission and during in-hospital stay	All participants with a smartphone
**Activity tracker**			
	Monitoring and giving feedback on recovery	Reducing uncertainties and fear related to the recovery process, which may improve recovery	All participants with a suitable smartphone for the activity tracker

###### Making a Personalized Convalescence Plan

A personalized and tailored convalescence plan, including advice about resumption of (work) activities is the most important tool on the website. The specific tailored convalescence recommendations were developed for relevant types of abdominal surgical procedures by using a modified Delphi procedure [53,54]. On the website, information will be tailored for each patient, offering the opportunity to enhance patient involvement. This is possible as some data are already prefilled when patients receive their website account (eg, surgical procedure, sex, and hospital). This tool will enhance recovery by giving clear and simple instructions for the RNA and will stop inconsistent recommendations from different health care providers.

###### Providing Information About the Surgical Procedure and Recovery Process

Information per treatment phase (preoperative, perioperative, and postoperative) will be provided by text and images. These services will contribute perioperative to the patients’ awareness and expectations; both factors have proven to be important predictors of the length of recovery. Information will also have a positive effect on anxiety and satisfaction because the patients can prepare themselves better for the surgery. Postoperative information will be offered about the recovery period and common postoperative complaints. This could support patients during this period and may help them with feelings of insecurity. In addition, practical advice about when, how, and whom to contact in case of complaints will be provided. Patients will be helped in deciding whether to contact a health care professional in case of complaints or complications during their recovery. Frequently asked questions will be added as well and these were formulated based on main topics in patients’ brochures and Web-based patient discussion forums. This tool will take away the insecurity with respect to the postoperative course, symptoms, and complications.

###### Getting Feedback on Recovery by a Recovery Monitor and Recovery Report

The recovery monitor and report are tools to identify recovery problems and give patients feedback on their recovery progress. Patients will be asked to indicate in a recovery monitor to what extent they have resumed their activities, which will be subsequently graphically displayed in a recovery report allowing them to track their progress. It also aims to improve monitoring and transition of postoperative care. After the patient has given consent, the Web portal can be accessed by a health care professional in secondary care to monitor patients’ recovery and, thus, identify recovery problems.

##### Developing the Materials and Tools of the Revised “ikherstel” Intervention

###### Providing Information on the Website

For patients who will receive adjuvant chemotherapy, information about this treatment, including side effects, is provided on the website. This will provide extra attention and mental support in case of patients with a malignant indication. Videos are also added on the website. Videos are considered the most appropriate medium to deliver an informative message to patients because of the influence of modeling behavior on attitude [55]. Videos about the admission day, the surgical procedures, receiving and managing a permanent or definitive stoma, the postoperative period, and a simulation of patients and employers to discuss potential RTW problems are provided.

###### Postoperative Consult by Electronic Consultation

In case of recovery problems, patients have the option to ask questions to a health care professional from their own hospital by means of an eConsult via the website. Patients will be informed that eConsults are only suitable for nonurgent questions and that these questions will be answered within 2 working days. In case of urgent questions, they receive a phone number for direct contact. The hypothesis is that patients will be more comfortable and less hampered in resuming their activities with the opportunity to ask questions whenever they prefer. This tool will let patients have more interaction with their health care professional via a Web portal.

##### Mobile Phone App (Mobile Health)

All information which is available on the website is also available on the mobile phone app (“ikherstel” app), which will be synchronized with the website. This includes among others the convalescence plan that patients created on the website. A section to make notes and the option to compose a list of what to pack when being admitted to the hospital will also be available on the app. If patients do not have a smartphone, they will only use the website. This tool will enhance recovery by giving clear and simple instructions for the RNA and will stop inconsistent recommendations from different health care providers (see Multimedia Appendix 4).

##### Activity Tracker

An activity tracker that measures the daily step count can be linked to the “ikherstel” application for patients with a smartphone. This tracker will be used as a support tool for patients to monitor and to give feedback on their recovery. The average daily step count in the week before surgery will be set as their baseline measurement and, thereby, target postoperative activity level. The daily step count will be postoperative graphically displayed in the app as a percentage of their target activity level, including a target level the patient is expected to reach. Patients will be asked to wear the activity tracker in the first 4 weeks after surgery, and again after 8 weeks as the hypothesis is that baseline activity level should be reached in this week. This tool will encourage patients to a quicker and more intense mobilization and earlier RNA.

##### Pretest of Materials

In total, 10 patients and a representative sample of health care professionals evaluated the demo version of the eHealth “ikherstel” intervention. Patients got 3 weeks to test the intervention before they were interviewed by the researcher. Semistructured interviews were conducted with patients who also had participated in the FGDs. Health care professionals were asked to judge the demo version on several items (eg, layout, comprehensibility of all informative text). The test patients were satisfied with the content of the information, the way it was delivered, and the messages (source and style). They also found all text provided on the website as very useful. However, a few remarks for improvement were suggested. These were related to supplying more detailed information about the side effects of chemotherapy, adding more attention to mental support, and less complicated sentences. All test patients would like to use the intervention if they will have surgery again.

#### Refinement and Production of Materials After Testing

After participants’ and health care professionals’ feedback, minimal adjustments were made. Mainly textual changes were made to simplify and order sentences. This resulted in the final eHealth intervention that was used to perform the RCT.

### Step 5: Program Implementation Plan

Patients undergoing major abdominal, general surgical, or gynecological procedures were identified as new program users. No new professionals were identified as program users as the introduction and implementation of the program would remain in the perioperative phase also for the extended patient group. The 7 hospitals that participated in the earlier performed “ikherstel” study wished to continue the use of the “ikherstel” intervention including broadening the usage of “ikherstel” intervention at the general surgery department of their hospital. Many other general hospitals in different regions in the Netherlands signed the letter of intent to participate in this multicenter study. After a kick-off meeting at the research institute, one meeting with surgeons or gynecologists (depending on the department) and one meeting with nurses per hospital was held to discuss the content and logistics of the study and its implementation. Finally, 10 general surgery practices and 8 gynecology practices (all teaching hospitals) were positive about the further development of “ikherstel” intervention and willing to implement the intervention. They will participate in the evaluation of this intervention through implementation of the eHealth intervention as a supplement to the standard perioperative care given at their hospital.

### Step 6: Evaluation

#### Study Design

The evaluation of the eHealth intervention will be performed by a multicenter single-blinded RCT. The effectiveness and cost-effectiveness of this innovative eHealth care program compared with the usual care given in 11 participating hospitals on RNA and RTW will be evaluated. A process evaluation will also be performed using a mixed-methods design [56].

#### Eligibility Criteria

Patients who will undergo a laparoscopic or open colectomy or hysterectomy and are aged between 18 and 75 years will be contacted. Exclusion criteria are surgery without a curative intention, deep infiltrating endometriosis, concomitant surgical procedures, not able to use the internet, unable to understand Dutch questionnaires, malignancy (in case of the hysterectomy), and receiving neoadjuvant treatment.

#### Outcome Measures

Our primary outcome measure is RNA. The Patient-Reported Outcomes Measurement Information System physical functioning item bank version 1.2 will be used to measure limitations in daily activities. A list of 29 most relevant selected activities will be presented to participants before surgery with the goal to select 8 activities, which are most relevant for them in their daily life. They will be asked in the following questionnaires after surgery if they can already perform one of these 8 activities. RNA is defined as the time in calendar days from the day of surgery until a participant has resumed all activities [57,58].

Secondary outcomes are social participation, self-rated health, duration until RTW, physical activity, length of recovery, pain intensity, and patient satisfaction [59-63]. Costs will be measured from a societal and health care perspective and consist of costs of the intervention, health care utilization costs, and costs associated with lost productivity [64,65]. Sociodemographic data and questions regarding expectations about the length of recovery and the amount of anxiety will be assessed at the baseline measurement. Complications will be assessed by reviewing the surgical reports and postoperative notes and scored by using the Clavien-Dindo classification [66]. The outcome measures will be obtained by using questionnaires administered at baseline (approximately 1-2 week preoperative) and at 2, 4, and 6 weeks and 3, 6, 9, and 12 months after surgery. For a total overview of all outcomes per measurement moment of this study, see Multimedia Appendix 5.

#### Sample Size Calculation

To detect a hazard ratio of 1.4 for RNA (corresponding to a decrease in median time to RNA from 10 weeks to 7.14 weeks as a result of the intervention) with 80% power while testing using a 2-sided log-rank test at a significance level of 5%, a total of 318 events need to be observed. The total sample size is set at 354 (177 per arm) to account for an anticipated proportion of 2.5% of patients not returning to daily activities within the 12 month follow-up period and a dropout rate of 10%.

#### Recruitment, Inclusion, Allocation, and Blinding of Patients

Patients will be recruited for study participation when they are on the waiting list in one of the participating hospitals and will receive a study information letter on behalf of their doctor. Contact will be made by phone to check their willingness to participate and to access eligibility. Eligible patients willing to participate will be included. After the patient completes the baseline questionnaire T(0) within 2 week before surgery and has signed informed consent, randomization will be executed by an (independent) research assistant. A computer-generated randomization in a 1:1 ratio will be performed on individual level stratified regarding hospital, sex, and surgical procedure using permuted blocks of size 2. Patients will be blinded to the intervention, as they do not know which program is developed as a nonintervention or intervention care program. The researchers involved in the analyses will be blinded to the allocation throughout the analyses. Health care professionals cannot be blinded to the intervention because it is highly likely that they will be notified of the allocation either by the patient or the patients’ medical file.

#### Data Analyses Plan

All analyses will be performed in IBM SPSS. Baseline characteristics will be summarized using descriptive statistics and compared between the intervention and control group using *t* tests by normal distributions of variables and Mann-Whitney *U* tests, chi-square tests, or Fisher exact tests by non-normal distributions of variables. Our primary outcome, time until full RNA, will be analyzed by both crude and adjusted survival analyses where hospital, surgical procedure, and sex will be taken into account as covariates in the adjusted analyses due to stratification. To describe the distribution of the duration until RNA in both groups, the Kaplan Meier method will be used. The Cox proportional hazard model will be applied to calculate hazard ratios. Adjustments will be made if there are clinically relevant differences between the intervention and the control group in the baseline characteristics or if other potential confounding factors are observed. For the longitudinal secondary outcomes, mixed models and multilevel logistic regression models will be performed. For the cross-sectional secondary outcomes, *t* tests, Mann-Whitney *U* tests, chi-square tests, or Fisher exact tests will be used to compare differences. Intention-to-treat analyses will be compared with per-protocol analyses to identify whether there are differences if patients used the intervention as intended. Subgroup analyses will be performed regarding the surgical procedure (colectomy and hysterectomy) and indication (benign or malignant disease). A post hoc analysis will be carried out on patients without major complications.

## Discussion

### Principal Findings

The purpose of this study was to describe the systematic process of the further development of the “ikherstel” intervention following the 6 steps of the IM protocol [20,21]. This eHealth intervention was adjusted to target different behavioral determinants relevant for the overarching program goal to achieve earlier full recovery for general surgical and gynecological patients. From the beginning, patients and health care professionals were involved in the developmental process and patients’ needs were taken into account by offering a user-friendly eHealth and mHealth intervention for our target group [67]. By involving hospitals in an early stage of the development process, the expectation is that a supportive basis for the intervention was created and that the implementation followed by evaluation will go as planned [68].

### Comparison With Other Studies

The IM protocol has proven to be suitable to systematically and scientifically develop an eHealth intervention for various health issues [69,70]. In addition, a few studies have also used the IM protocol to further develop, adapt, or adjust an existing intervention [71,72]. In these studies, the IM protocol was considered suitable and enabled researchers to reconsider points of view and to integrate new information into existing tools. These studies used the IM protocol in the same manner as we did in this study. They decided to further build on the methodological choices made in the previously developed intervention by adjusting and improving the existing intervention for a new study population [71,72]. Methodological choices made in steps 2 and 3 of the IM process of developing the original “ikherstel” intervention, as described in the IM article of Vonk Noordegraaf et al, are considered effective and, therefore, used again in this IM procedure [19].

Comparison with other studies focusing on the development of eHealth recovery interventions is limited due to a low number of studies [18,23]. When comparing this developed intervention to the eHealth interventions included in the systematic review conducted by our planning group in step 1, it can be concluded that our intervention consists of multiple components on a diversity of functions that makes this a comprehensive intervention. In addition, most RCTs regarding enhancing the postoperative course are performed in cardiac surgery, whereas there is a lack of RCTs evaluating postoperative recovery after general surgical and gynecological procedures [73-80]. The studies of Vonk et al and Bouwsma et al are most comparable [18,23]. However, our study will be the only RCT that will be performed in patients undergoing major abdominal surgery focusing on RNA, which also includes procedures performed due to a malignant indication [81]. By determining the time until RNA as primary outcome, all patients aged between 18 and 75 years can participate in this study allowing more people to be reached.

The intervention was further developed and improved with respect to the previous “ikherstel” intervention. The shortcomings identified in the needs assessment are reflected, and new tools have been introduced. On the basis of the input from the FGDs, the accessibility has been improved by developing a mobile app and by the possibility of an eConsult. This facilitates direct access and communication with health care professionals and increases the reach of the “ikherstel” intervention [82]. By using the activity tracker, patients can monitor themselves, resulting in more involvement in their recovery process and motivating them to mobilize quicker and more intensively. Given the “ikherstel” intervention is based on the concept of computer-driven tailoring, the information regarding recovery is more personally relevant, which will increase the likelihood of behavior change and maintenance [83,84].

### Strengths and Limitations

One of the strengths of our study is that by involving patients and different health care professionals in the development process of the intervention, experiences from multiple fields of expertise were included. We are convinced that this is necessary because this recovery-focused eHealth intervention is designed to include a multidisciplinary approach. Another strength is that all information gathered in the development (IM) process of the original intervention was based on findings of qualitative and quantitative studies and is used and extended with additional information obtained during this IM process. By adjusting and improving the “ikherstel” intervention, a more complete eHealth intervention for the broadened target population was created.

A limitation is that nurses were not involved in this process of development of the intervention, whereas they are linked to a part of the intervention itself. However, we did have meetings with nurses in step 5 of the IM protocol, which attributes to the acceptability.

### Clinical Relevance

Involving different program users and using a theory- and evidence-based systematic approach in the development of the intervention as noted in the IM procedure results in the best opportunity on effectiveness and implementation. If the further developed “ikherstel” intervention is proven effective, the content can be extended to other surgical procedures. In addition, if it proves to be cost-effective for a broad surgical group of patients, an implementation plan for future nationwide implementation has to be generated. It will also provide insight into the question of whether a systematically further developed version of an effective intervention is still effective for a different target group.

### Conclusions

This study showed that with the use of IM, we were able to optimize and further develop the original “ikherstel” intervention. The intervention is extended to patients undergoing general surgical procedures and for malignant indications. New intervention tools such as a mobile app, an activity tracker, and an eConsult were developed. Consequently, with these tools, there is an increase in accessibility coupled with provision of monitoring and interactive feedback. The further developed “ikherstel” intervention will be evaluated in a multicenter single-blinded RCT.

## Appendix

Multimedia Appendix 1 Performance objectives to empower patients to return to normal activities including work. Asterisk indicates that the performance objective is based on the intervention mapping study of Vonk Noordegraaf et al (2014).

Multimedia Appendix 2 Example of performance objective of patients.

Multimedia Appendix 3 Determinants, methods, preconditions and strategies tools.

Multimedia Appendix 4 The layout of the mobile app (directly translated from Dutch).

Multimedia Appendix 5 Primary and secondary outcomes per measurement moment.

## Abbreviations

eConsult: electronic consultation
eHealth: electronic health
ERAS: enhanced recovery after surgery programs
FGD: focus group discussion
ICT: information and communication technology
IM: Intervention Mapping
mHealth: mobile health
PRECEDE: predisposing, reinforcing, and enabling constructs in educational diagnosis and evaluation
PROCEED: policy, regulatory, and organizational constructs in educational and environmental development
RCT: randomized controlled trial
RNA: resumption of normal activities
RTW: return to work
