# Supplementary figures and images for: Electronic Health Program to Empower Patients in Returning to Normal Activities After General Surgical and Gynecological Procedures: Intervention Mapping as a Useful Method for Further Development
Source: J Med Internet Res. 2019 Feb 6;21(2):e9938. doi: 10.2196/jmir.9938 (PMC6381532; doi:10.2196/jmir.9938)

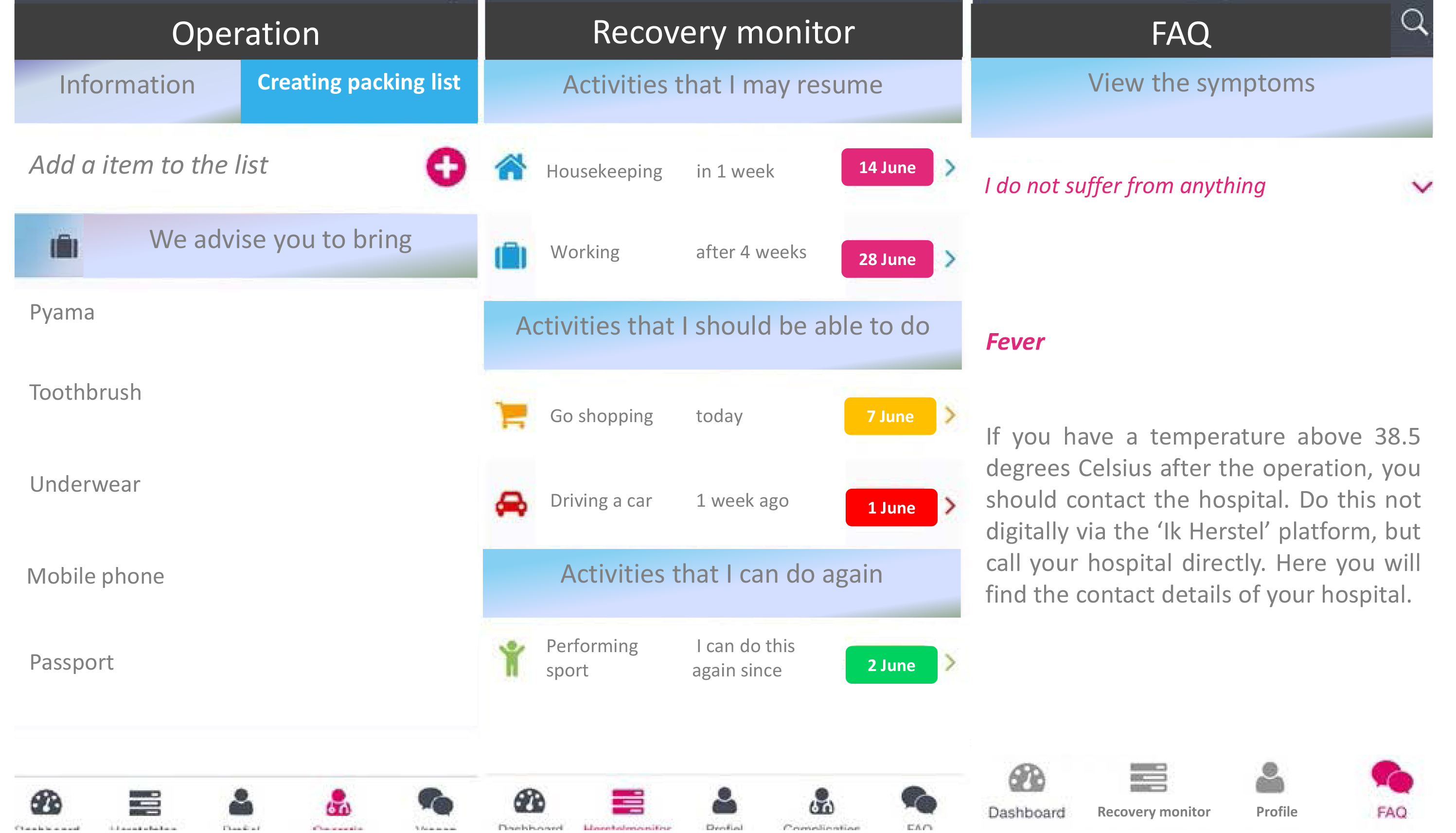

Supplement: Multimedia Appendix 4 [file jmir_v21i2e9938_app4.png]
